# Beeswax as Dental Filling on a Neolithic Human Tooth

**DOI:** 10.1371/journal.pone.0044904

**Published:** 2012-09-19

**Authors:** Federico Bernardini, Claudio Tuniz, Alfredo Coppa, Lucia Mancini, Diego Dreossi, Diane Eichert, Gianluca Turco, Matteo Biasotto, Filippo Terrasi, Nicola De Cesare, Quan Hua, Vladimir Levchenko

**Affiliations:** 1 Multidisciplinary Laboratory, “Abdus Salam” International Centre for Theoretical Physics, Trieste, Italy; 2 Centre for Archaeological Science, University of Wollongong, Wollongong, New South Wales, Australia; 3 Department of Environmental Biology, University “La Sapienza”, Rome, Italy; 4 Sincrotrone Trieste S.C.p.A., AREA Science Park, Basovizza (Trieste), Italy; 5 Department of Medical Sciences, University of Trieste, Trieste, Italy; 6 CIRCE, INNOVA and Department of Environmental Sciences, 2nd University of Naples, Caserta, Italy; 7 CIRCE, INNOVA and Department of Life Sciences, 2nd University of Naples, Caserta, Italy; 8 Australian Nuclear Science and Technology Organisation, Lucas Heights, New South Wales, Australia; Museo Nazionale Preistorico Etnografico ‘L. Pigorini’, Italy

## Abstract

Evidence of prehistoric dentistry has been limited to a few cases, the most ancient dating back to the Neolithic. Here we report a 6500-year-old human mandible from Slovenia whose left canine crown bears the traces of a filling with beeswax. The use of different analytical techniques, including synchrotron radiation computed micro-tomography (micro-CT), Accelerator Mass Spectrometry (AMS) radiocarbon dating, Infrared (IR) Spectroscopy and Scanning Electron Microscopy (SEM), has shown that the exposed area of dentine resulting from occlusal wear and the upper part of a vertical crack affecting enamel and dentin tissues were filled with beeswax shortly before or after the individual’s death. If the filling was done when the person was still alive, the intervention was likely aimed to relieve tooth sensitivity derived from either exposed dentine and/or the pain resulting from chewing on a cracked tooth: this would provide the earliest known direct evidence of therapeutic-palliative dental filling.

## Introduction

Several molar crowns with regularly shaped cavities with concentric ridges, discovered some six years ago in a Neolithic graveyard in Pakistan, are the most ancient evidence of dentistry practice [1]. Other findings that suggest dental interventions during the Neolithic are very rare and include a very dubious therapeutic dental treatment identified in the Gaione graveyard (Italy) [2] and an artificial tooth from the cemetery of Gebel Ramlah (Egypt), which could have been used as a dental prosthesis [3], [4].

Although the possibility of treatment of sensitive tooth structure by means of some type of filling has been supposed [1], there is no published evidence, as far as we know, on the use of therapeutic-palliative substances in prehistoric dentistry. In ancient Egypt, external applications, composed of honey mixed with mineral ingredients, were used to fix loose teeth or to reduce the pain, as reported in the Papyrus Ebers, dating back to the XVI century BC [5].

In this emerging framework of ancient dental therapeutic practices, the finding of a human partial mandible associated with contemporary beeswax, covering the occlusal surface of a canine, could represent a possible case of therapeutic use of beeswax during the Neolithic.

The interpretation of the evidence obtained in this study, based on the use of advanced analytical methods, supports the hypothesis of an intentional therapeutic treatment, but alternative post-mortem practices are not ruled out.

## Materials and Methods

**Figure 1:**
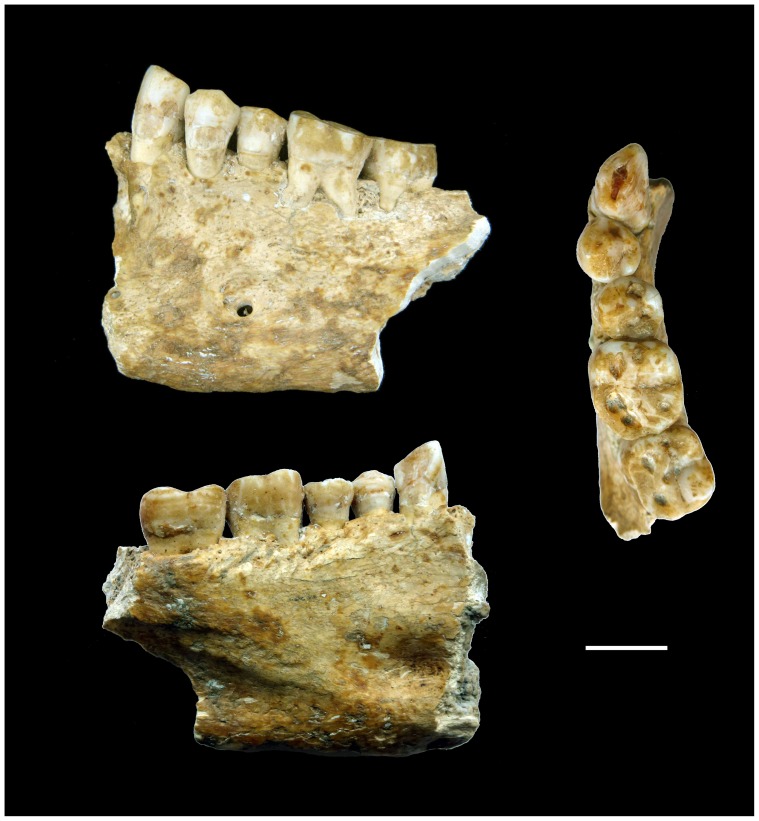
The Lonche jaw from a karstic cave of southern Slovenia. Scale bar, 10 mm.

The specimen here described (Lonche 1), kept in the Natural History Museum of Trieste, Italy, consists of the left portion of an isolated adult mandible bearing a canine, two premolars, and the first two molars (Figure 1). The mandible was found partially embedded in calcite on the wall of a karstic cave near the village of Lonche, in northern Istria (Slovenia). It was discovered associated to some Upper Pleistocene fauna remains and was consequently considered one of the most ancient anthropological remains from the northern-Adriatic area. However, with the exception of a short and not really informative note published soon after the discovery [6], a detailed description of the sample and archaeological context is scanty or not available.

On the external aspect of the *corpus,* the mental foramen is located under the fourth premolar and the oblique line is well marked and extends beneath the second molar, while the ramus ends just before the third molar. On its internal aspect, under the well expressed mylohyoid line, the specimen exhibits deep and anteroposteriorly elongated submandibular and sublingual fossae. A remnant of the digastric fossa is still preserved just before the anterior fracture. The degree of occlusal wear, assessed following Lovejoy (1985), likely indicates that Lonche 1 represents a 24–30-year-old individual (phase E) [7]. The preserved morphology of the mental region, the thickness of the mandibular body, and the dental crown size suggest more likely a male sex diagnosis. A pronounced enamel hypoplasia is present on the cervical half of the buccal aspect of the canine and premolars as well as on that of the lingual aspect of the second molar.

Parts of the enamel are broken in the cervical half of the buccal aspect of the canine and the third premolar probably due to taphonomic factors.

Radiocarbon analysis, performed by accelerator mass spectrometry (AMS) on collagen extracted from the mandibular bone, has provided an age range of 6655-6400 cal. BP (2σ), which corresponds to the Neolithic in northern Istria and, in particular, to the post-Vlaška phase. The area, at the northern shore of the Adriatic Sea, is rich in archaeological cave sites and rock shelters, many of which were occupied during recent prehistory and mainly used for stabling animals. In fact, the Neolithic economy of the karstic area was mainly based on sheep breeding [8]. The neolithization process spread through northern Istria and Trieste Karst in the middle of the 6^th^ millennium BC, coming from the south along the eastern Adriatic coast, and is referred to as the so-called Vlaška Culture. This culture shows connections with the Danilo Culture of central Dalmatia and the first Neolithic sites of the Friuli plain and lasts up to the beginning of the 5^th^ millennium BC. The post-Vlaška assemblages (5^th^ millennium BC) are not yet well understood and a clear chrono-cultural sequence is not available. It seems that there was no substantial change with respect to previous pottery shapes, some of which disappeared [9], [10]. However, a few and new typological elements suggest cultural affinities with Dalmatia (Hvar Culture) and northern Italy (Square Mouthed Pottery Culture).

### Micro-CT Analyses

**Figure 2:**
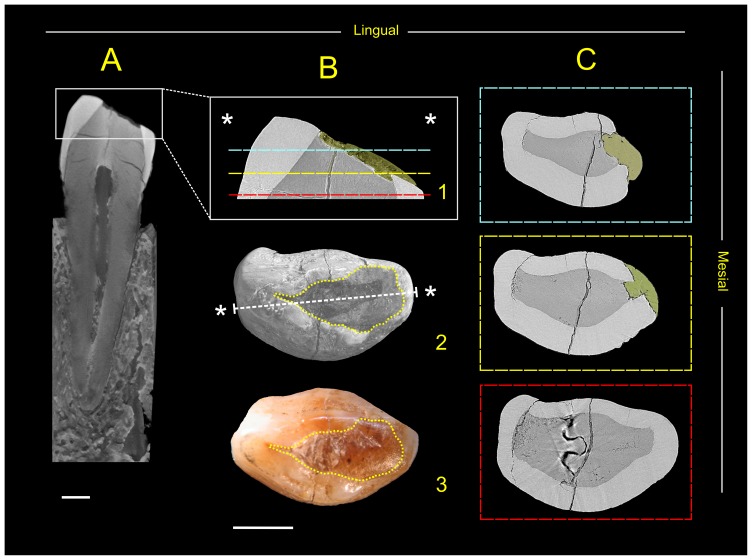
The Lonche canine. A) Distal-mesial virtual section of the entire Lonche 1 lower left canine (resolution 18 µm). B) 1. Micro-CT detail of the crown showing the thickness of the beeswax (in yellow). Beeswax exactly fills the shallow cavity in the exposed dentin and the upper part of the crack (resolution 9 µm). 2. Three-dimensional virtual reconstruction and 3. microphotograph of the tooth crown in occlusal view with indication of the surface covered by beeswax (within the yellow dotted line). C) Micro-CT based cross-sections of the tooth showing the enamel cracks along the labial and lingual aspects (resolution 9 µm). The positions of the cross-sections are shown in Figure B2. The beeswax is colored in yellow. Scale bars, 2 mm.

The entire canine was analysed by X-ray micro-CT at the TOMOLAB, located at the Elettra Synchrotron Light Laboratory in Trieste (Italy) (Figure 2A). TOMOLAB is based on a microfocus X-ray source (minimum focal spot size 5 µm, voltage up to 130 kV). A water-cooled, 12 bit, 4008x2672 pixels CCD camera, with a maximum active area of 50×33 mm^2^, was used as a detector. Exploiting the cone beam geometry a complete reconstruction of the object with 18 µm isotropic voxel size has been obtained. The micro-CT scan was carried out with a source voltage of 130 kV, a current of 61 µA and recording 2400 projections of the sample over 360 degrees.

The upper part of the left canine was analysed with synchrotron radiation micro-CT at the SYRMEP beamline of Elettra, using phase-contrast enhanced imaging based on a free-space propagation approach (Figure 2B–C). A 12 bit, water-cooled CCD camera with an active area of 18×12 mm^2^ and a pixel size of 9 µm was used as a detector. The scan was performed by using a monochromatic X-ray beam, at an energy of 35 keV, with the sample-to-detector distance of 500 mm and recording 1200 projections of the sample over 180 degrees.

For the cone-beam slice reconstruction the software COBRA (EXXIM Computing Corporation), GPU-based, has been used. Synchrotron based micro-CT slices have been reconstructed by using the Syrmep_tomo_project 4.0 software, custom-developed at Elettra.

**Figure 3:**
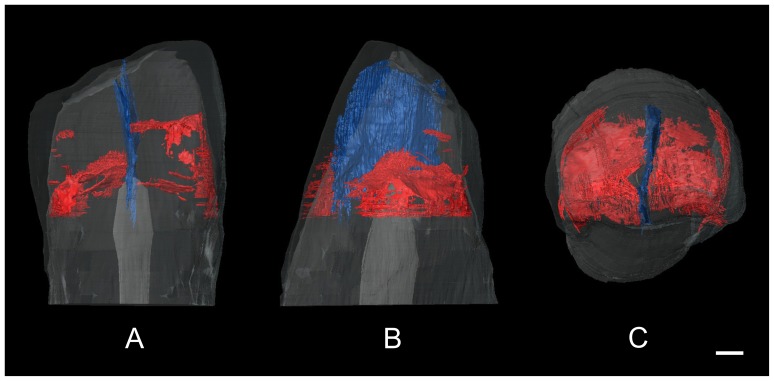
Volume rendering of the canine crown showing the fractures through the dental tissues in transparency. The main vertical fracture (in blue) and the sub-horizontal ones (in red) are shown in lingual (A), distal (B) and occlusal view (C). Scale bar, 1 mm.

The volume renderings have been obtained using VGStudio max 2.0 while the segmentation and volume renderings of the canine and its cracks have been obtained using Amira 5.3 (Figure 3).

### IR Analysis of the Filling Material

**Figure 4:**
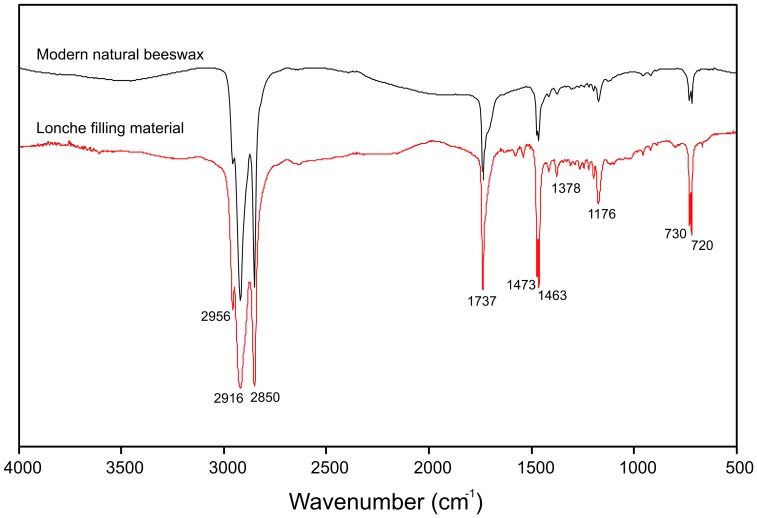
Comparison between the IR spectrum of Lonche filling material and a modern sample of natural beeswax. The Lonche spectrum is an average of 10 analyses.

A small volume (<2 mm^3^) of the filling material was extracted with tweezers tips and the sample was squeezed into a diamond anvil cell before analysis to obtain a very thin and homogeneous layer. The sample was analysed in transmission mode using a Perkin Elmer Spectrum Spotlight 200 microscope attached to a Spectrum 100 spectrometer (black body source, MCT detector) at the Restoration Centre, Institute for the Protection of Cultural Heritage of Slovenia (ZVKDS). For statistical purposes and relevance, 10 different positions of the sample were analysed in the 4000 cm^−1^ – 500 cm^−1^ spectral range, with a resolution of 4 cm^−1^ and 128 accumulations per spectrum (Figure 4).

### AMS Radiocarbon Dating of the Lonche Jaw

The bone sample was collected using a conventional hand drill. Collagen extraction was carried out using the following chemical procedures [11]:

About 1 g of bone powder was treated twice with HCl (0.6 N) at room temperature to remove apatite (carbonate and phosphates), preserving the humic fraction as solid phase.
The acid-insoluble component was then treated with NaOH (0.1 N) for a short time to remove base-soluble contaminants such as humic acids. After this alkali treatment, further HCl (0.6N) was used to remove dissolved CO_2_ from the sample. Several rinses with deionized water were used after each reagent.
The collagen extracted was dried in the oven overnight.

After the above chemical pre-treatment, the collagen was combusted in a sealed pre-cleaned quartz tube with copper oxide in grains via muffle furnace combustion for 6.5 hr at 920°C. The CO_2_ produced by the combustion was purified into a steel cryogenic line through H_2_O and CO_2_ spiral traps and transferred to a sealed pre-cleaned pyrex tube with Zn and TiH_2_ powder where the graphitization took place at 565°C for 8 hr [12].

Finally, the resulting graphite was pressed into an aluminum cathode and measured by means of the CIRCE AMS system [13].

**Figure 5:**
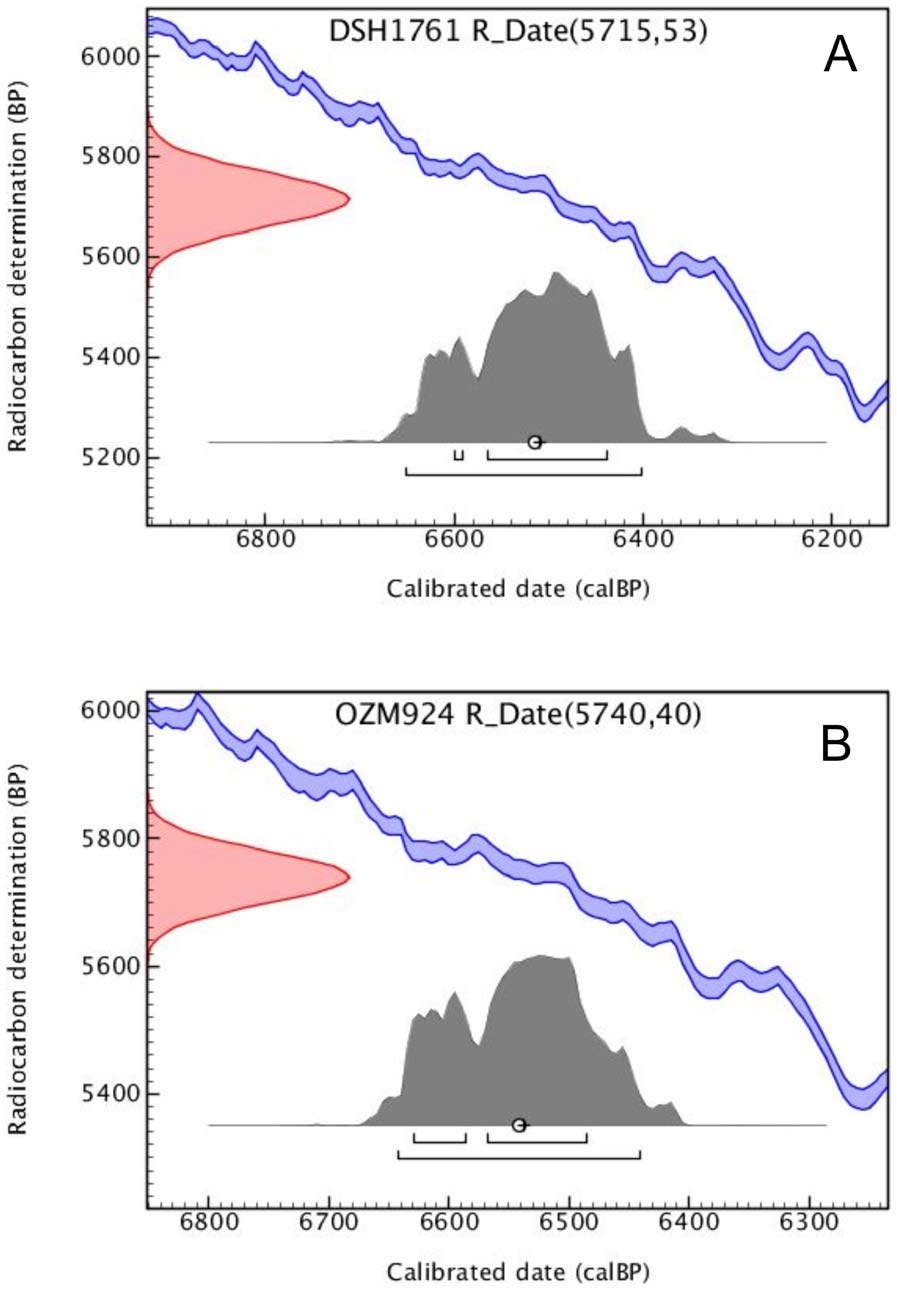
Calibration of radiocarbon dates of the Lonche jaw and beeswax. A) Calibration of a single radiocarbon date (DSH1761 = jaw) using the IntCal09 data set [15] and OxCal program v.4.1.3 [14]. B) Calibration of a single radiocarbon date (OZM924 = beeswax) using the IntCal09 data set [15] and OxCal program v.4.1.3 [14]. Blue lines depict the IntCal09 calibration curve (shown at 1σ range). Red curve indicates the Gaussian distribution of the radiocarbon date. Grey histogram represents the probability distribution of the calibrated age. Black lines depict calibrated age ranges for 1σ and 2σ. Open circle and cross represent weighted mean and median, respectively.

The radiocarbon age obtained, reported in Table 1 as DSH1761 code, was calibrated using the OxCal v 4.1.3 program [14], considering the IntCal09 calibration curve [15] (Figure 5).

**Table 1:** Calibrated ages of the Lonche jaw and the beeswax.

Lab code	Conventional ^14^C ages (BP)	Calibrated ages (cal BP)						
		68.2% or 1-sigma		95.4% or 2-sigma				
		from	to	from	to	mean	sigma	median
DSH1761	5715±53	6600	6435	6655	6400	6515	70	6510
OZM924	5740±40	6630	6485	6645	6440	6540	55	6540

DSH1761 is the sample from the jaw and OZM924 is the beeswax. The dates have been calibrated using the IntCal09 dataset [15] and OxCal program v.4.1.3 [14].

### AMS Radiocarbon Dating of the Beeswax Sample

Beeswax was extracted from the solid sample using high-purity diethyl ether (99.9% pure, spectrophotometric grade, inhibitor-free) from Sigma-Aldrich. The sample (2.3 mg) was placed into a thimble, made from a clean Whatman GF/A glass microfibre filter, which was then placed into a micro-soxhlet connecting to a condenser at the upper end and to a 25 ml conical flask containing 20 ml of diethyl ether at the lower end. This set-up was placed on a hot plate and in a fume cupboard. Solvent extraction was carried out for two hours and the temperature of the micro-soxhlet was kept at ∼60°C during this process.

After the extraction the volume of solvent was reduced to ∼5 ml by gently heating the conical flask with a hair dryer. The solvent was then transferred into a pre-cleaned Vycor® silica combustion tube at ∼2 ml a time and diethyl ether was evaporated using a hair dryer. After most of the diethyl ether was removed, the combustion tube was attached to a vacuum line. The tube was evacuated for one hour then heated with a hair dryer for ten minutes under vacuum. The evacuation continued for another hour. Finally, the combustion tube containing the beeswax sample was oven-dried overnight at 60°C, which is well above the boiling point of diethyl ether of 34.6°C, to make sure that the sample was free of diethyl ether.

The beeswax sample of 1.4 mg was combusted to CO_2_ in the presence of pre-cleaned CuO and Ag wires using the sealed-tube technique, and converted to graphite using the H_2_/Fe method. The technical aspects of these methods have been described in Hua et al. [16]. The graphite mass was 1.26 mg. A small portion of graphite was employed for the determination of δ^13^C using the Micromass IsoPrime Elemental Analyser/Isotope Ratio Mass Spectrometer (EA/IRMS) at ANSTO. AMS ^14^C measurements were performed using the STAR facility at ANSTO [17].

The result is reported as conventional radiocarbon age after correction for measured δ^13^C for isotopic fractionation, and presented in Table 1 as OZM924 code. Calibrated ^14^C age at 2σ (95.4% confidence level) was calculated using the IntCal09 data set [15] and the calibration program OxCal v 4.1.3 [14] (Figure 5).

### SEM of the Occlusal Surface

**Figure 6:**
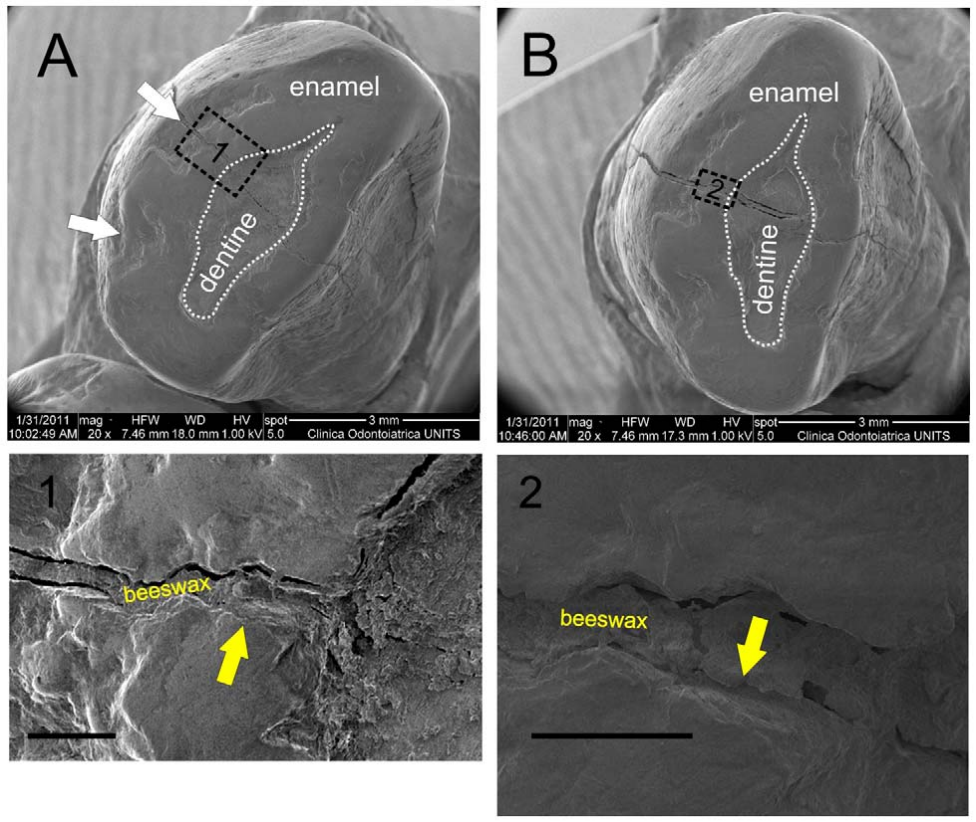
SEM images of the occlusal surface of the Lonche canine after the beeswax was removed. The Figures A and B, respectively taken before and after cleaning the occlusal surfaces from beeswax superficial residues, show the exposed area of dentine resulting from occlusal wear and the vertical crack still filled with beeswax. Some chippings with round and smooth edges, indicated by the white arrows in Figure A, are present on the occlusal buccal margin of tooth. In Figure A1 residues of beeswax cover the edges of the vertical crack, while Figure B2 shows that some enamel fragments are lost in the same area, indicated by yellow arrows. Scale bars, 200 µm.

The occlusal surface of the canine was observed by means of a Quanta250 SEM (FEI, Oregon, USA) operating in secondary electron detection mode at the Department of Medical Sciences of University of Trieste. Increasing enlargement magnifications from 50–1000x were used. The working distance was 10 mm and the accelerating voltage was 1kV (Figure 6).

Some images have been taken after the removal of the beeswax for the radiocarbon dating without cleaning the occlusal surface (Figure 6A); other images have been acquired after an accurate cleaning, to observe the morphology of the occlusal surface and to check the possible presence of some kind of dental intervention (Figure 6B).

We obtained permission from the Natural History Museum of Trieste to study the Lonche mandible, which was originally donated to the Museum by the finders.

## Results

Direct radiocarbon analyses of the mandible and of the dental filling, performed by AMS, has provided an age range of 6655-6400 cal. BP (2σ) and 6645-6440 cal. BP (2σ) respectively, demonstrating that the dental filling covers the canine occlusal surface since Neolithic times.

The micro-CT analyses and the segmentation of all the fractures inside the tooth (Figure 3) show that a vertical crack, noticeable on the buccal and lingual outer enamel (Figure 2C), also affected the inner enamel and the coronal dentin, extending to the top of the pulp chamber (Figure 2A; Figure 3). It also shows that the extraneous material covers an exposed area of dentine resulting from occlusal wear and penetrates the crack for 1.43 mm (Figure 2B-C).

Between the top of the pulp cavity and the occlusal worn dentin basin, the vertical crack passes through a radio-dense longitudinal region, which could correspond to a sclerosed dead tract as a response of wear (Figure 2A). The sclerotic dentin is in fact characterised by higher density induced by the hypermineralization of peritubular and intertubular zones and an obliteration of the dentin tubules [18].

Other cracks present in the tooth’s crown show a completely different orientation. These damages radiate horizontally from the central axis of the crown, developing from the vertical crack without crossing it. Most of these cracks stop in correspondence of the dentin-enamel junction (DEJ) or run through it (Fig. 2A; Figure 3).

The 10 IR spectra obtained from different areas of the sample are similar and can be easily superimposed, only the global intensity of the signal, due to the amount of material crossed by the infrared beam, changes. No difference in the band position or its relative proportion can be noticed and therefore no discrepancy in the signal exists.

Intense ν_C-H_ stretching bands at 2956, 2916 and 2850 cm^−1^ and δC-H bending vibrations in the 1475-1377 cm^−1^ domain can be attributed to the presence of an aliphatic compound. Stretch vibrations of CH_2_ bonds lead to high absorption at 2916 and 2850 cm^−1^ with a small band at 2956 cm^−1^ due to CH_3_. Sharp doublets at 1473 and 1463 cm^−1^, indicating CH_2_ bending vibrations, have also been observed. Rocking vibrations at 730 and 720 cm^−1^ result from the out of plane deformation of successive methylene groups and suggest a compound with a long aliphatic chain (but also n-alkanes and esters present in beeswax). This is confirmed by the relatively low intensity of the ν_C–H_ asymmetric stretching and δ_C–H_ symmetric bending band of methyl group at 2956 and 1378 cm^−1^, respectively. In addition, a progression of absorption bands is noticed in the 1350-1200 cm^−1^ region. They are assigned to wagging and twisting vibrations of successive methylene groups which are coupled to a carboxyl group and indicate the presence of acyl lipids and lipids with a hydroxyl groups. The number of these bands was previously attributed to the presence of palmitic acid. The composition of this lipid compound is, furthermore, identified from its ester component, *i.e.* a wax ester, which presents a ν_C = O_ stretching band at 1737 cm^−1^, and confirmed by the ν_C–O_ stretching bands at 1176 and 1113 cm^−1^. In addition, vibrations in the 1600-1500 cm^−1^ domain indicate the presence of fatty acid salts, also confirmed by an absorption band at 1713 cm^−1^ representative of a fatty acid or at least of a carboxylic acid. The two peaks at 1579 and 1541 cm^−1^ indicate that calcium carboxylates constitute a small part of the filling component. These salts could be formed by a reaction of free fatty acids with mineral compound, which is consistent with the composition of the tooth, based on calcium phosphate minerals. Moreover, the aging of the material is indicated by the fact that the ester could have been partially converted into carboxylic acid and alcohol due to oxidation phenomena, present at 1713 and 1015 cm^−1^, respectively.

The features of the IR spectra indicate a composition of the tooth filling material that matches better beeswax (Figure 4), while other natural waxes can give similar bands but not in the same proportion and ratio between bands [19]–[23].

The SEM analysis of the occlusal surface shows that the beeswax not only filled the exposed area of dentine resulting from occlusal wear but also penetrated in the longitudinal crack. This buccolingual fracture runs through modest chippings present on the occlusal buccal margin of tooth. A post-depositional origin of the chippings is very unlikely since their margins are very smooth and round (indicated by white arrows in Figure 6A).

Moreover, the observation of the edges of the crack before and after its cleaning gives information to assess *antemortem/perimortem* or *postmortem* origin of the fracture possibly caused by changes in temperature and humidity.

The edges of the fracture are generally not rounded but some small chippings are present on the fracture margin. In one area, which was covered by beeswax (indicated by yellow arrows in Figure 6), the edges of the fracture have different shapes indicating that some enamel fragments have been lost producing a stair-like profile (Figure 6B2). Since the chippings along the fracture were sealed by the beeswax and no tooth fragments have been collected during the cleaning of the occlusal surface of the canine, the chippings occurred during the Neolithic before the application of the beeswax probably due to the crack formation process or the masticatory use of the tooth.

## Discussion

The discovery of beeswax filling in the Lonche canine can have an acceptable explanation. This substance has already been reported to have been used as a binding agent during antiquity [23], [24]. Furthermore, thanks to its extreme chemical stability [19], being composed of long-chain wax esters, unsaturated and saturated n-alkanes, diesters and hydroxyesters [25], beeswax can be preserved for long periods of time.

On the other hand, the origin of the vertical crack needs to be discussed in detail since the possible *antemortem/perimortem* or *postmortem* nature of the trauma implies quite different interpretations for the presence of beeswax on the tooth. There are only a few archaeological reports on ancient human tooth injuries [26]–[28] but quite abundant literature on fracture types and their propagation in human teeth is available. According to one of the most recent classifications of tooth fractures in living individuals, the crack can be defined as a fracture in vertical plane in an anterior tooth where the fracture line passes buccolingually in the crown (Type 3, Div 2, A) [29].

Hughes *et al.* [30] have proposed a method to differentiate between *antemortem/perimortem* from *postmortem* trauma based on the different behavior of dentin and enamel tissues during dehydration processes since enamel *in vivo* has a much lower percentage of water than dentin. Postmortem damages are characterised by propagation of the cracks from the dentin, due to water loss and subsequent shrinking, through the DEJ and out toward the enamel.

On the other hand, the propagation patterns of *in vivo* damages, when a high compressive stress is applied to the exterior tooth surface, initiate from the enamel to the DEJ and the mantle dentin, which generally dissipates a large part of the forces or even arrests the propagation of the crack [30], [31]. However, some cracks can penetrate the dentin as demonstrated by clinical reports [29].

The cracks radiating horizontally from the central axis of the Lonche crown, ending near the dentin-enamel junction (DEJ) and running along it, correspond to the description of *postmortem* fractures by Hughes *et al.* [30] with the exception that the cracks start from the longitudinal axes of the tooth and not from the pulp chamber (Figure 3). On the other hand the morphology of the vertical crack, running from the occlusal surface to the tip of the pulp chamber (Figure 3), is compatible with both *antemortem/perimortem* and *postmortem* models. (Figure 2) [30].

Moreover, the conclusions drawn by Hughes *et al.* are limited by the small number of samples, the use of pig teeth for the analysis since the human ones have slightly different structure and mechanical properties [30], no testing of *antemortem* contexts such as the possible *in vivo* exposure to temperature fluctuation and the effects of *perimortem* trauma [30] or dental wear, which is particularly significant for the studied specimen.

Three main hypotheses, implying different timings in the vertical crack formation, can be introduced to explain the beeswax deposition on the occlusal surface of the Lonche canine: a) the exposed area of dentine and the vertical crack were filled *in vivo* with beeswax; b) the beeswax was deposited *in vivo* on the exposed area of dentine but the crack opened up post-mortem and drew beeswax into it; c) the beeswax could have been placed on the tooth after the death of the individual and the crack could have or not already been developed at that time. Such a postmortem intervention could be related to secondary burial practices, which, however, are completely unknown in northern Istria.

Although the second and third hypotheses cannot be completely ruled out, in our opinion several elements make the antemortem hypothesis of the crack formation more convincing.

1. First of all, the SEM images indicate that the beeswax was probably deposited on the tooth when the crack was already formed since the chippings on the edges of the fracture were sealed by the beeswax (Figure 6).
2. Chippings of the enamel on the buccal margin of the occlusal surface (Figure 6A) indicate that the tooth was subjected to compressive external stresses, which could have also originated the vertical fracture. Such pronounced dental wear is common in Neolithic remains, often reflecting diet and extramasticatory use of teeth [32].
3. The fracture passes through a radio-dense longitudinal region, which probably corresponds to a sclerosed dead-tract as a response to wear. The crack occurred after the sclerosed dentin had formed in the axial plane of the crown beneath the worn dentin because it would have killed the pulp. Since the hardness and elasticity of sclerosed dentin is much lower than vital one [18], a potential crack would have preferentially propagated through the sclerosed tissues.
4. Also other teeth (Figure 1) have exposed dentin but no beeswax was applied. This suggests that the canine caused particular discomfort during life. Concerning a possible postmortem application of the beeswax, one could wonder why it was applied only on the exposed dentin of the canine.

Due to the exposed dentin and possibly the vertical crack, the tooth probably became very sensitive, limiting the functionality of the jaw during occlusion. The occlusal surface could have been filled with beeswax in an attempt to reduce the pain sealing exposed dentin tubules and the fracture from changes in osmotic pressure (as occurs on contact with sugar) and temperature (hot or cold relative to the oral cavity). The binding properties of beeswax could have been increased by the probable presence of honey, one of the main ingredients of external applications used in ancient Egypt to fix loose teeth or to reduce the tooth pain [5]. Traces of beeswax, detected in a few prehistoric pottery vessels from Britain, have in fact been interpreted as possible honey residues [33].

If this hypothesis is correct, as no obvious periapical reaction is detectable, either the individual died shortly after the event, as suggested also by the little rounding of the fracture edges, or else, if he survived, the tooth evidently progressively lost its functionality without experiencing any infection, swelling of the pulp or bone loss (Figure 2A).

The discovery of propolis pellets preserved among the grave goods in some late Upper Paleolithic and Mesolithic burials of northeastern Italy [34]–[36] testifies that hunter**-**gatherers were already using resinous aromatic bee products, suitable also for therapeutic-palliative purposes. Bee products were largely used by prehistoric communities for technological, artistic and medical purposes [33], [37]–[41] but here we report, for the first time, its possible use for therapeutic-palliative dental filling.
